# Supplementary material for: End Sequence Analysis Toolkit (ESAT) expands the extractable information from single-cell RNA-seq data
Source: Genome Res. 2016 Oct;26(10):1397–410. doi: 10.1101/gr.207902.116 (PMC5052061; doi:10.1101/gr.207902.116)
Supplement: Supplemental Material [file supp_gr.207902.116_Supp_Materials.tar.gz › ESAT-esat_v0.1/doc/html/classbroad_1_1pda_1_1seq_1_1segmentation_1_1_continuous_data_alignment_model.html]

ESAT: broad.pda.seq.segmentation.ContinuousDataAlignmentModel Class Reference


ESAT


- Main Page
- Related Pages
- Packages
- Classes
- Files

- Class List
- Class Index
- Class Hierarchy
- Class Members

All Classes Namespaces Files Functions Variables Enumerator Pages

- broad
- pda
- seq
- segmentation
- ContinuousDataAlignmentModel

Public Member Functions |
Static Public Member Functions |
Static Public Attributes |
List of all members

broad.pda.seq.segmentation.ContinuousDataAlignmentModel Class Reference

Collaboration diagram for broad.pda.seq.segmentation.ContinuousDataAlignmentModel:

[legend]

|  |  |
| --- | --- |
| Public Member Functions | |
|  | ContinuousDataAlignmentModel (AlignmentDataModel data) throws IOException |
|  | |
|  | ContinuousDataAlignmentModel (AlignmentDataModelStats data) throws IOException |
|  | |
|  | ContinuousDataAlignmentModel (AlignmentDataModelStats data, int EF, int minSpliceSupport) throws IOException |
|  | |
|  | ContinuousDataAlignmentModel (AlignmentDataModelStats data, File[] maskFiles, int minSpliceSupport) throws IOException |
|  | |
|  | ContinuousDataAlignmentModel (AlignmentDataModelStats data, File[] maskFiles, int EF, int minSpliceSupport, AlignmentDataModelStats pairedData, AlignmentDataModelStats strandSpecificReads) throws IOException |
|  | |
|  | ContinuousDataAlignmentModel (AlignmentDataModelStats data, File[] maskFiles, int EF, int minSpliceSupport, AlignmentDataModelStats pairedData, AlignmentDataModelStats strandSpecificReads, boolean upweight) throws IOException |
|  | |
|  | ContinuousDataAlignmentModel (AlignmentDataModelStats data, Map< String, Integer > maskFileData, int EF, int minSpliceSupport, AlignmentDataModelStats pairedData, AlignmentDataModelStats strandSpecificReads) throws IOException |
|  | |
|  | ContinuousDataAlignmentModel (AlignmentDataModelStats data, Map< String, Integer > maskFileData, int EF, int minSpliceSupport, AlignmentDataModelStats pairedData, AlignmentDataModelStats strandSpecificReads, boolean upWeight) throws IOException |
|  | |
|  | ContinuousDataAlignmentModel (AlignmentDataModelStats data, Map< String, Integer > maskFileData, int EF, int minSpliceSupport, AlignmentDataModelStats pairedData, AlignmentDataModelStats strandSpecificReads, int chunkSize, boolean upWeightSplices) throws IOException |
|  | |
|  | ContinuousDataAlignmentModel (AlignmentDataModelStats data, File[] maskFiles, int EF, int minSpliceSupport, AlignmentDataModelStats pairedData) throws IOException |
|  | |
|  | ContinuousDataAlignmentModel (AlignmentDataModelStats data, File[] maskFiles, int EF, int minSpliceSupport) throws IOException |
|  | |
| void | setFindMaxContiguous (boolean findMaxContiguous) |
|  | |
| void | setTrimEnds (boolean trimEnds) |
|  | |
| void | setMinContguousSegmentSize (int minSize) |
|  | |
| void | setAlpha (double alpha) |
|  | |
| void | setUpWeightSplices (boolean setFlag) |
|  | |
| void | setMinNumberOfSplices (int minSpliceSupport) |
|  | |
| void | setExtensionFactor (int extensionFactor) |
|  | |
| void | setMaskFileData (Map< String, Integer > maskedRegionMap) |
|  | |
| void | setMinimumMappingQuality (double minMapQual) |
|  | |
| void | setTrimQuantile (double quantile) |
|  | |
| AlignmentDataModelStats | getData () |
|  | |
| double | getLambda (String chr) throws IOException |
|  | |
| double | getSum (String chr) throws IOException |
|  | |
| double | getNumberOfReads (String chr) throws IOException |
|  | |
| double | getNumberMarkers (String chr) throws IOException |
|  | |
| double | getNumberOfBPs () |
|  | |
| int | getCount (Collection< Annotation > Annotation) throws IOException |
|  | |
| int[] | getGeneCounts (Collection< Gene > genes) throws IOException |
|  | |
| int[] | getGeneCountsWithoutDatasetTotals (Collection< Gene > genes) throws IOException |
|  | |
| double[] | getCountsPerBp (Annotation align, IntervalTree< Alignment > tree) |
|  | |
| List< Double > | getCountsPerBp (Gene gene, IntervalTree< Alignment > tree) |
|  | |
| Map< Gene, double[]> | getGeneExpression (Collection< Gene > genes) throws IOException |
|  | |
| int | getCounts (Map< String, IntervalTree< Annotation >> geneTree, Collection< String > multimappers) throws IOException |
|  | |
| int | getChromosomeLength (String chr) |
|  | |
| Map< String, Integer > | getChromosomeLengths () |
|  | |
| double[] | getDataForAlignment (Annotation align) throws IOException |
|  | |
| List< Double > | getDataForGene (Gene gene) throws IOException |
|  | |
| Map< Gene, List< Double > > | getDataForGene (Collection< Gene > Annotation) throws IOException |
|  | |
| AlignmentDataModelStats | getPairedData () |
|  | |
| Collection< Annotation > | scan (int[] windowSizes, double alpha) throws IOException |
|  | |
| Collection< Annotation > | scan (int[] windowSizes, double alpha, String chr) throws IOException |
|  | |
| void | scanGenome (ContinuousDataAlignmentModel data2, int windowSize, String chr, FileWriter writer, boolean filterSignificance) throws IOException |
|  | |
| double[] | scanPRate (Gene gene) throws IOException |
|  | |
| double[] | scanPRate (Gene gene, IntervalTree< Alignment > tree) throws IOException |
|  | |
| double[] | scanPRate (Gene gene, IntervalTree< Alignment > tree, double localLambda) throws IOException |
|  | |
| double[] | scanPRate (Gene gene, IntervalTree< Alignment > tree, double localLambda, int numMarkers) throws IOException |
|  | |
| double[] | scanRegion (int fixedWidth, LightweightGenomicAnnotation region) throws IOException |
|  | |
| double[] | scoreSegment (Annotation align) throws IOException |
|  | |
| Map< Annotation, double[]> | scoreSegments (Collection< Annotation > set) throws IOException |
|  | |
| Map< Annotation, double[]> | scoreSegments (Collection< Annotation > set, String chrToUse) throws IOException |
|  | |
| Map< Annotation, double[]> | scoreSegments (Collection< Annotation > set, String chrToUse, boolean ignoreAlpha) throws IOException |
|  | |
| double[] | scoreGene (Gene align) throws IOException |
|  | |
| double[] | scoreGene (Gene align, double localLambda) throws IOException |
|  | |
| GeneScore | scoreGene (Gene align, IntervalTree< Alignment > tree, double localLambda) throws IOException |
|  | |
| GeneScore | scoreGene (Gene align, IntervalTree< Alignment > tree, double localLambda, int numMarkers) throws IOException |
|  | |
| Map< Gene, double[]> | scoreGenes (Collection< Gene > set, String chrToUse, IntervalTree< Alignment > tree) throws IOException |
|  | |
| GeneScore | getPeak (Gene gene, Gene startCodon, IntervalTree< Alignment > tree) throws IOException |
|  | |
| Gene | getPeak (Gene gene, Gene startCodon) throws IOException |
|  | |
| AlignmentDataModelStats | getAlignmentDataModelStats () |
|  | |
| double[] | scoreGene (Gene align, IntervalTree< Alignment > tree) throws IOException |
|  | |
| Map< Gene, double[]> | scoreGenes (Collection< Gene > genes) throws IOException |
|  | |
| Map< Gene, double[]> | scoreGenes (Collection< Gene > set, String chrToUse) throws IOException |
|  | |
| Map< Gene, double[]> | scoreGenes (Collection< Gene > set, String chrToUse, int size) throws IOException |
|  | |
| void | scoreGenes (BEDFileParser genes) throws IOException |
|  | |
| Map< Path, double[]> | scorePathsForOnlyRPKM (Collection< Path > paths) |
|  | |
| Map< Path, double[]> | scorePaths (Collection< Path > paths, double lambda) throws IOException |
|  | |
| double | count (Annotation align) throws IOException |
|  | |
| Gene | trimMaxContiguous (Gene region, double quantile) throws IOException |
|  | |
| List< Gene > | filterGenesByExpression (Map< Gene, List< Double >> countsMap) |
|  | |
| Gene | trimGeneEnds (Gene gene, double quantile) throws IOException |
|  | |
| void | writeConnections (String save, ChromosomeWithBubblesJGraphT graph) throws IOException |
|  | |
| boolean | hasDataForChromosome (String chr) |
|  | |
| void | resetTreeCache () |
|  | |
| double | CV (Gene gene, IntervalTree< Alignment > tree) throws IOException |
|  | |
| Collection< Annotation > | permuteRegions (Annotation align) throws IOException |
|  | |
| Collection< Annotation > | rawRegions (Annotation align) throws IOException |
|  | |
| int | getBasesCovered (Annotation region) throws IOException |
|  | |
| int | getBasesCovered (Annotation region, int EF) throws IOException |
|  | |

|  |  |
| --- | --- |
| Static Public Member Functions | |
| static int[] | getWidths (String str) |
|  | |
| static Map< Path, double[]> | scorePaths (Collection< Path > paths, ChromosomeWithBubblesJGraphT graph, double alpha) |
|  | |
| static Map< Path, double[]> | scorePaths (Collection< Path > paths, double lambda, double numMarkers, double numReads, double RPKMContant, Map< Annotation, Double > localRates, double alpha) |
|  | |
| static double | calculatePVal (int k, double lambda, double w, double T) |
|  | |
| static void | writeFullBED (String save, Map< Gene, double[]> map, double alpha) throws IOException |
|  | |
| static void | writeFullBED (String save, Collection< Gene > segments) throws IOException |
|  | |
| static void | main (String[] args) throws Exception |
|  | |
| static Collection< Gene > | filterPaths (Map< Path, double[]> scores, double alpha2) |
|  | |
| static Map< String, Integer > | parseMaskFiles (File[] files) throws IOException |
|  | |

|  |  |
| --- | --- |
| Static Public Attributes | |
| static int | DEFAULT\_MIN\_MAPPING\_QUALITY = 5 |
|  | |
| static int | DEFAULT\_INSERT\_SIZE\_FUDGE = 20 |
|  | |
| static double | DEFAULT\_INS\_SIZE\_PVAL = 0.05 |
|  | |
| static double | DEFAULT\_MAKE\_GENE\_OVERLAP = 0.3 |
|  | |

## Constructor & Destructor Documentation

|  |  |  |  |  |  |
| --- | --- | --- | --- | --- | --- |
| broad.pda.seq.segmentation.ContinuousDataAlignmentModel.ContinuousDataAlignmentModel | ( | AlignmentDataModel | *data* | ) | throws IOException |

Constructor to build the data alignment model

Parameters
:   |  |  |
    | --- | --- |
    | data | the alignment data model that stores the read data |

Exceptions
:   |  |  |
    | --- | --- |
    | IOException |  |

Here is the caller graph for this function:

|  |  |  |  |  |  |
| --- | --- | --- | --- | --- | --- |
| broad.pda.seq.segmentation.ContinuousDataAlignmentModel.ContinuousDataAlignmentModel | ( | AlignmentDataModelStats | *data* | ) | throws IOException |

Constructor to build the data alignment model

Parameters
:   |  |  |
    | --- | --- |
    | data | the alignment data model that computes the statistics and stores the read data |

Exceptions
:   |  |  |
    | --- | --- |
    | IOException |  |

|  |  |  |  |
| --- | --- | --- | --- |
| broad.pda.seq.segmentation.ContinuousDataAlignmentModel.ContinuousDataAlignmentModel | ( | AlignmentDataModelStats | *data*, |
|  |  | int | *EF*, |
|  |  | int | *minSpliceSupport* |
|  | ) |  | throws IOException |

Constructor to build the data alignment model

Parameters
:   |  |  |
    | --- | --- |
    | data | the alignment data model that computes the statistics and stores the read data |
    | EF | an extension factor used to extend reads |
    | minSpliceSupport | the minimum number of spliced reads needed to add a connection in the graph |

Exceptions
:   |  |  |
    | --- | --- |
    | IOException |  |

|  |  |  |  |
| --- | --- | --- | --- |
| broad.pda.seq.segmentation.ContinuousDataAlignmentModel.ContinuousDataAlignmentModel | ( | AlignmentDataModelStats | *data*, |
|  |  | File[] | *maskFiles*, |
|  |  | int | *minSpliceSupport* |
|  | ) |  | throws IOException |

Constructor to build the data alignment model

Parameters
:   |  |  |
    | --- | --- |
    | data | the alignment data model that computes the statistics and stores the read data |
    | maskFiles | the mask files |
    | minSpliceSupport | the minimum number of spliced reads needed to add a connection in the graph |

Exceptions
:   |  |  |
    | --- | --- |
    | IOException |  |

|  |  |  |  |
| --- | --- | --- | --- |
| broad.pda.seq.segmentation.ContinuousDataAlignmentModel.ContinuousDataAlignmentModel | ( | AlignmentDataModelStats | *data*, |
|  |  | File[] | *maskFiles*, |
|  |  | int | *EF*, |
|  |  | int | *minSpliceSupport*, |
|  |  | AlignmentDataModelStats | *pairedData*, |
|  |  | AlignmentDataModelStats | *strandSpecificReads* |
|  | ) |  | throws IOException |

Constructor to build the data alignment model

Parameters
:   |  |  |
    | --- | --- |
    | data | the alignment data model that computes the statistics and stores the read data |
    | maskFiles | the mask files |
    | EF | an extension factor used to extend reads |
    | minSpliceSupport | the minimum number of spliced reads needed to add a connection in the graph |
    | pairedData | the alignment data model that stores the stats for paired end reads and the paired end read data |
    | strandSpecificReads | an alignment data model for the strand specific reads |

Exceptions
:   |  |  |
    | --- | --- |
    | IOException |  |

Here is the call graph for this function:

|  |  |  |  |
| --- | --- | --- | --- |
| broad.pda.seq.segmentation.ContinuousDataAlignmentModel.ContinuousDataAlignmentModel | ( | AlignmentDataModelStats | *data*, |
|  |  | File[] | *maskFiles*, |
|  |  | int | *EF*, |
|  |  | int | *minSpliceSupport*, |
|  |  | AlignmentDataModelStats | *pairedData*, |
|  |  | AlignmentDataModelStats | *strandSpecificReads*, |
|  |  | boolean | *upweight* |
|  | ) |  | throws IOException |

Constructor to build the data alignment model

Parameters
:   |  |  |
    | --- | --- |
    | data | the alignment data model that computes the statistics and stores the read data |
    | maskFiles | the mask files |
    | EF | an extension factor used to extend reads |
    | minSpliceSupport | the minimum number of spliced reads needed to add a connection in the graph |
    | pairedData | the alignment data model that stores the stats for paired end reads and the paired end read data |
    | strandSpecificReads | an alignment data model for the strand specific reads |
    | chunkSize | the size of the cache chunk used |

Exceptions
:   |  |  |
    | --- | --- |
    | IOException |  |

Here is the call graph for this function:

|  |  |  |  |
| --- | --- | --- | --- |
| broad.pda.seq.segmentation.ContinuousDataAlignmentModel.ContinuousDataAlignmentModel | ( | AlignmentDataModelStats | *data*, |
|  |  | Map< String, Integer > | *maskFileData*, |
|  |  | int | *EF*, |
|  |  | int | *minSpliceSupport*, |
|  |  | AlignmentDataModelStats | *pairedData*, |
|  |  | AlignmentDataModelStats | *strandSpecificReads* |
|  | ) |  | throws IOException |

Constructor to build the data alignment model

Parameters
:   |  |  |
    | --- | --- |
    | data | the alignment data model that computes the statistics and stores the read data |
    | maskFileData | a map of all unalignable regions of the genome |
    | EF | an extension factor used to extend reads |
    | minSpliceSupport | the minimum number of spliced reads needed to add a connection in the graph |
    | pairedData | the alignment data model that stores the stats for paired end reads and the paired end read data |
    | strandSpecificReads | an alignment data model for the strand specific reads |

Exceptions
:   |  |  |
    | --- | --- |
    | IOException |  |

|  |  |  |  |
| --- | --- | --- | --- |
| broad.pda.seq.segmentation.ContinuousDataAlignmentModel.ContinuousDataAlignmentModel | ( | AlignmentDataModelStats | *data*, |
|  |  | Map< String, Integer > | *maskFileData*, |
|  |  | int | *EF*, |
|  |  | int | *minSpliceSupport*, |
|  |  | AlignmentDataModelStats | *pairedData*, |
|  |  | AlignmentDataModelStats | *strandSpecificReads*, |
|  |  | boolean | *upWeight* |
|  | ) |  | throws IOException |

Constructor to build the data alignment model

Parameters
:   |  |  |
    | --- | --- |
    | data | the alignment data model that computes the statistics and stores the read data |
    | maskFileData | a map of all unalignable regions of the genome |
    | EF | an extension factor used to extend reads |
    | minSpliceSupport | the minimum number of spliced reads needed to add a connection in the graph |
    | pairedData | the alignment data model that stores the stats for paired end reads and the paired end read data |
    | strandSpecificReads | an alignment data model for the strand specific reads |
    | upWeightSplices | whether to weight spliced reads based on their proportioncoverage scores |

Exceptions
:   |  |  |
    | --- | --- |
    | IOException |  |

|  |  |  |  |
| --- | --- | --- | --- |
| broad.pda.seq.segmentation.ContinuousDataAlignmentModel.ContinuousDataAlignmentModel | ( | AlignmentDataModelStats | *data*, |
|  |  | Map< String, Integer > | *maskFileData*, |
|  |  | int | *EF*, |
|  |  | int | *minSpliceSupport*, |
|  |  | AlignmentDataModelStats | *pairedData*, |
|  |  | AlignmentDataModelStats | *strandSpecificReads*, |
|  |  | int | *chunkSize*, |
|  |  | boolean | *upWeightSplices* |
|  | ) |  | throws IOException |

Constructor to build the data alignment model

Parameters
:   |  |  |
    | --- | --- |
    | data | the alignment data model that computes the statistics and stores the read data |
    | maskFileData | a map of all unalignable regions of the genome |
    | EF | an extension factor used to extend reads |
    | minSpliceSupport | the minimum number of spliced reads needed to add a connection in the graph |
    | pairedData | the alignment data model that stores the stats for paired end reads and the paired end read data |
    | strandSpecificReads | an alignment data model for the strand specific reads |
    | chunkSize | the size of the cache chunk used |
    | upWeightSplices | whether to weight spliced reads based on their proportion when computing coverage scores |

Exceptions
:   |  |  |
    | --- | --- |
    | IOException |  |

|  |  |  |  |
| --- | --- | --- | --- |
| broad.pda.seq.segmentation.ContinuousDataAlignmentModel.ContinuousDataAlignmentModel | ( | AlignmentDataModelStats | *data*, |
|  |  | File[] | *maskFiles*, |
|  |  | int | *EF*, |
|  |  | int | *minSpliceSupport*, |
|  |  | AlignmentDataModelStats | *pairedData* |
|  | ) |  | throws IOException |

Constructor to build the data alignment model

Parameters
:   |  |  |
    | --- | --- |
    | data | the alignment data model that computes the statistics and stores the read data |
    | maskFiles | the mask files |
    | EF | an extension factor used to extend reads |
    | minSpliceSupport | the minimum number of spliced reads needed to add a connection in the graph |
    | pairedData | the alignment data model that stores the stats for paired end reads and the paired end read data |

Exceptions
:   |  |  |
    | --- | --- |
    | IOException |  |

|  |  |  |  |
| --- | --- | --- | --- |
| broad.pda.seq.segmentation.ContinuousDataAlignmentModel.ContinuousDataAlignmentModel | ( | AlignmentDataModelStats | *data*, |
|  |  | File[] | *maskFiles*, |
|  |  | int | *EF*, |
|  |  | int | *minSpliceSupport* |
|  | ) |  | throws IOException |

Constructor to build the data alignment model

Parameters
:   |  |  |
    | --- | --- |
    | data | the alignment data model that computes the statistics and stores the read data |
    | maskFiles | the mask files |
    | EF | an extension factor used to extend reads |
    | minSpliceSupport | the minimum number of spliced reads needed to add a connection in the graph |

Exceptions
:   |  |  |
    | --- | --- |
    | IOException |  |

## Member Function Documentation

|  |  |  |  |
| --- | --- | --- | --- |
| static double broad.pda.seq.segmentation.ContinuousDataAlignmentModel.calculatePVal | ( | int | *k*, |
|  |  | double | *lambda*, |
|  |  | double | *w*, |
|  |  | double | *T* |
|  | ) |  |  |

static

Here is the caller graph for this function:

|  |  |  |  |  |  |
| --- | --- | --- | --- | --- | --- |
| double broad.pda.seq.segmentation.ContinuousDataAlignmentModel.count | ( | Annotation | *align* | ) | throws IOException |

Here is the caller graph for this function:

|  |  |  |  |
| --- | --- | --- | --- |
| double broad.pda.seq.segmentation.ContinuousDataAlignmentModel.CV | ( | Gene | *gene*, |
|  |  | IntervalTree< Alignment > | *tree* |
|  | ) |  | throws IOException |

|  |  |  |  |  |  |
| --- | --- | --- | --- | --- | --- |
| List<Gene> broad.pda.seq.segmentation.ContinuousDataAlignmentModel.filterGenesByExpression | ( | Map< Gene, List< Double >> | *countsMap* | ) |  |

This function will filter the genes in the input Map by a number of constraints on the total number of reads on the genes

1. All genes with expression < 5.0 read counts will be removed
2. All genes with expression in the 75th quantile of the remaining expressed genes will be returned

   Parameters
   :   |  |  |
       | --- | --- |
       | countsMap | A map of RefSeqGene to a List of counts along the length of the gene |

   Returns

|  |  |  |  |
| --- | --- | --- | --- |
| static Collection<Gene> broad.pda.seq.segmentation.ContinuousDataAlignmentModel.filterPaths | ( | Map< Path, double[]> | *scores*, |
|  |  | double | *alpha2* |
|  | ) |  |  |

static

|  |  |  |  |  |
| --- | --- | --- | --- | --- |
| AlignmentDataModelStats broad.pda.seq.segmentation.ContinuousDataAlignmentModel.getAlignmentDataModelStats | ( |  | ) |  |

|  |  |  |  |  |  |
| --- | --- | --- | --- | --- | --- |
| int broad.pda.seq.segmentation.ContinuousDataAlignmentModel.getBasesCovered | ( | Annotation | *region* | ) | throws IOException |

Here is the caller graph for this function:

|  |  |  |  |
| --- | --- | --- | --- |
| int broad.pda.seq.segmentation.ContinuousDataAlignmentModel.getBasesCovered | ( | Annotation | *region*, |
|  |  | int | *EF* |
|  | ) |  | throws IOException |

Here is the call graph for this function:

|  |  |  |  |  |  |
| --- | --- | --- | --- | --- | --- |
| int broad.pda.seq.segmentation.ContinuousDataAlignmentModel.getChromosomeLength | ( | String | *chr* | ) |  |

Here is the caller graph for this function:

|  |  |  |  |  |
| --- | --- | --- | --- | --- |
| Map<String, Integer> broad.pda.seq.segmentation.ContinuousDataAlignmentModel.getChromosomeLengths | ( |  | ) |  |

Here is the caller graph for this function:

|  |  |  |  |  |  |
| --- | --- | --- | --- | --- | --- |
| int broad.pda.seq.segmentation.ContinuousDataAlignmentModel.getCount | ( | Collection< Annotation > | *Annotation* | ) | throws IOException |

|  |  |  |  |
| --- | --- | --- | --- |
| int broad.pda.seq.segmentation.ContinuousDataAlignmentModel.getCounts | ( | Map< String, IntervalTree< Annotation >> | *geneTree*, |
|  |  | Collection< String > | *multimappers* |
|  | ) |  | throws IOException |

Here is the call graph for this function:

|  |  |  |  |
| --- | --- | --- | --- |
| double [] broad.pda.seq.segmentation.ContinuousDataAlignmentModel.getCountsPerBp | ( | Annotation | *align*, |
|  |  | IntervalTree< Alignment > | *tree* |
|  | ) |  |  |

Here is the caller graph for this function:

|  |  |  |  |
| --- | --- | --- | --- |
| List<Double> broad.pda.seq.segmentation.ContinuousDataAlignmentModel.getCountsPerBp | ( | Gene | *gene*, |
|  |  | IntervalTree< Alignment > | *tree* |
|  | ) |  |  |

|  |  |  |  |  |
| --- | --- | --- | --- | --- |
| AlignmentDataModelStats broad.pda.seq.segmentation.ContinuousDataAlignmentModel.getData | ( |  | ) |  |

Get the underlying AlignmentDataModelStats object

Returns
:   the AlignmentDataModelStats object

Here is the caller graph for this function:

|  |  |  |  |  |  |
| --- | --- | --- | --- | --- | --- |
| double [] broad.pda.seq.segmentation.ContinuousDataAlignmentModel.getDataForAlignment | ( | Annotation | *align* | ) | throws IOException |

This function returns an array of type double with counts for each base pair across the genomic region of the gene

Author
:   skadri

Parameters
:   |  |  |
    | --- | --- |
    | align |  |

Returns

Exceptions
:   |  |  |
    | --- | --- |
    | IOException |  |

Here is the call graph for this function:

|  |  |  |  |  |  |
| --- | --- | --- | --- | --- | --- |
| List<Double> broad.pda.seq.segmentation.ContinuousDataAlignmentModel.getDataForGene | ( | Gene | *gene* | ) | throws IOException |

Here is the call graph for this function:

Here is the caller graph for this function:

|  |  |  |  |  |  |
| --- | --- | --- | --- | --- | --- |
| Map<Gene, List<Double> > broad.pda.seq.segmentation.ContinuousDataAlignmentModel.getDataForGene | ( | Collection< Gene > | *Annotation* | ) | throws IOException |

Here is the call graph for this function:

|  |  |  |  |  |  |
| --- | --- | --- | --- | --- | --- |
| int [] broad.pda.seq.segmentation.ContinuousDataAlignmentModel.getGeneCounts | ( | Collection< Gene > | *genes* | ) | throws IOException |

|  |  |  |  |  |  |
| --- | --- | --- | --- | --- | --- |
| int [] broad.pda.seq.segmentation.ContinuousDataAlignmentModel.getGeneCountsWithoutDatasetTotals | ( | Collection< Gene > | *genes* | ) | throws IOException |

|  |  |  |  |  |  |
| --- | --- | --- | --- | --- | --- |
| Map<Gene, double[]> broad.pda.seq.segmentation.ContinuousDataAlignmentModel.getGeneExpression | ( | Collection< Gene > | *genes* | ) | throws IOException |

|  |  |  |  |  |  |
| --- | --- | --- | --- | --- | --- |
| double broad.pda.seq.segmentation.ContinuousDataAlignmentModel.getLambda | ( | String | *chr* | ) | throws IOException |

Get an estimate of the expected read coverage per base

Parameters
:   |  |  |
    | --- | --- |
    | chr | chromosome to retrieve |

Returns
:   lambda

Exceptions
:   |  |  |
    | --- | --- |
    | IOException |  |

Here is the caller graph for this function:

|  |  |  |  |  |  |
| --- | --- | --- | --- | --- | --- |
| double broad.pda.seq.segmentation.ContinuousDataAlignmentModel.getNumberMarkers | ( | String | *chr* | ) | throws IOException |

Gets the number of allowable bases per chr

Parameters
:   |  |  |
    | --- | --- |
    | chr | chromosome to retrieve |

Returns
:   total number of allowable bases

Here is the caller graph for this function:

|  |  |  |  |  |
| --- | --- | --- | --- | --- |
| double broad.pda.seq.segmentation.ContinuousDataAlignmentModel.getNumberOfBPs | ( |  | ) |  |

Get total number of bases

Returns
:   total number of bases

|  |  |  |  |  |  |
| --- | --- | --- | --- | --- | --- |
| double broad.pda.seq.segmentation.ContinuousDataAlignmentModel.getNumberOfReads | ( | String | *chr* | ) | throws IOException |

Gets the total number of reads per chr

Parameters
:   |  |  |
    | --- | --- |
    | chr | chromosome to retrieve |

Returns
:   total number of reads

Here is the call graph for this function:

|  |  |  |  |  |
| --- | --- | --- | --- | --- |
| AlignmentDataModelStats broad.pda.seq.segmentation.ContinuousDataAlignmentModel.getPairedData | ( |  | ) |  |

|  |  |  |  |
| --- | --- | --- | --- |
| GeneScore broad.pda.seq.segmentation.ContinuousDataAlignmentModel.getPeak | ( | Gene | *gene*, |
|  |  | Gene | *startCodon*, |
|  |  | IntervalTree< Alignment > | *tree* |
|  | ) |  | throws IOException |

|  |  |  |  |
| --- | --- | --- | --- |
| Gene broad.pda.seq.segmentation.ContinuousDataAlignmentModel.getPeak | ( | Gene | *gene*, |
|  |  | Gene | *startCodon* |
|  | ) |  | throws IOException |

|  |  |  |  |  |  |
| --- | --- | --- | --- | --- | --- |
| double broad.pda.seq.segmentation.ContinuousDataAlignmentModel.getSum | ( | String | *chr* | ) | throws IOException |

Gets the total number of reads per chr

Parameters
:   |  |  |
    | --- | --- |
    | chr | chromosome to retrieve |

Returns
:   total number of reads

Here is the caller graph for this function:

|  |  |  |  |  |  |
| --- | --- | --- | --- | --- | --- |
| static int [] broad.pda.seq.segmentation.ContinuousDataAlignmentModel.getWidths | ( | String | *str* | ) |  |

static

Here is the caller graph for this function:

|  |  |  |  |  |  |
| --- | --- | --- | --- | --- | --- |
| boolean broad.pda.seq.segmentation.ContinuousDataAlignmentModel.hasDataForChromosome | ( | String | *chr* | ) |  |

|  |  |  |  |  |  |
| --- | --- | --- | --- | --- | --- |
| static void broad.pda.seq.segmentation.ContinuousDataAlignmentModel.main | ( | String[] | *args* | ) | throws Exception |

static

Here is the call graph for this function:

|  |  |  |  |  |  |
| --- | --- | --- | --- | --- | --- |
| static Map<String, Integer> broad.pda.seq.segmentation.ContinuousDataAlignmentModel.parseMaskFiles | ( | File[] | *files* | ) | throws IOException |

static

Here is the caller graph for this function:

|  |  |  |  |  |  |
| --- | --- | --- | --- | --- | --- |
| Collection<Annotation> broad.pda.seq.segmentation.ContinuousDataAlignmentModel.permuteRegions | ( | Annotation | *align* | ) | throws IOException |

|  |  |  |  |  |  |
| --- | --- | --- | --- | --- | --- |
| Collection<Annotation> broad.pda.seq.segmentation.ContinuousDataAlignmentModel.rawRegions | ( | Annotation | *align* | ) | throws IOException |

|  |  |  |  |  |
| --- | --- | --- | --- | --- |
| void broad.pda.seq.segmentation.ContinuousDataAlignmentModel.resetTreeCache | ( |  | ) |  |

Here is the caller graph for this function:

|  |  |  |  |
| --- | --- | --- | --- |
| Collection<Annotation> broad.pda.seq.segmentation.ContinuousDataAlignmentModel.scan | ( | int[] | *windowSizes*, |
|  |  | double | *alpha* |
|  | ) |  | throws IOException |

|  |  |  |  |
| --- | --- | --- | --- |
| Collection<Annotation> broad.pda.seq.segmentation.ContinuousDataAlignmentModel.scan | ( | int[] | *windowSizes*, |
|  |  | double | *alpha*, |
|  |  | String | *chr* |
|  | ) |  | throws IOException |

Here is the call graph for this function:

|  |  |  |  |
| --- | --- | --- | --- |
| void broad.pda.seq.segmentation.ContinuousDataAlignmentModel.scanGenome | ( | ContinuousDataAlignmentModel | *data2*, |
|  |  | int | *windowSize*, |
|  |  | String | *chr*, |
|  |  | FileWriter | *writer*, |
|  |  | boolean | *filterSignificance* |
|  | ) |  | throws IOException |

Here is the call graph for this function:

|  |  |  |  |  |  |
| --- | --- | --- | --- | --- | --- |
| double [] broad.pda.seq.segmentation.ContinuousDataAlignmentModel.scanPRate | ( | Gene | *gene* | ) | throws IOException |

|  |  |  |  |
| --- | --- | --- | --- |
| double [] broad.pda.seq.segmentation.ContinuousDataAlignmentModel.scanPRate | ( | Gene | *gene*, |
|  |  | IntervalTree< Alignment > | *tree* |
|  | ) |  | throws IOException |

|  |  |  |  |
| --- | --- | --- | --- |
| double [] broad.pda.seq.segmentation.ContinuousDataAlignmentModel.scanPRate | ( | Gene | *gene*, |
|  |  | IntervalTree< Alignment > | *tree*, |
|  |  | double | *localLambda* |
|  | ) |  | throws IOException |

|  |  |  |  |
| --- | --- | --- | --- |
| double [] broad.pda.seq.segmentation.ContinuousDataAlignmentModel.scanPRate | ( | Gene | *gene*, |
|  |  | IntervalTree< Alignment > | *tree*, |
|  |  | double | *localLambda*, |
|  |  | int | *numMarkers* |
|  | ) |  | throws IOException |

|  |  |  |  |
| --- | --- | --- | --- |
| double [] broad.pda.seq.segmentation.ContinuousDataAlignmentModel.scanRegion | ( | int | *fixedWidth*, |
|  |  | LightweightGenomicAnnotation | *region* |
|  | ) |  | throws IOException |

|  |  |  |  |  |  |
| --- | --- | --- | --- | --- | --- |
| double [] broad.pda.seq.segmentation.ContinuousDataAlignmentModel.scoreGene | ( | Gene | *align* | ) | throws IOException |

Parameters
:   |  |  |
    | --- | --- |
    | align |  |

Returns
:   {calculatePVal(new Double(sum).intValue(), localLambda, geneLength, getNumberMarkers(chr)), enrich, sum, avgCoverage, rpkm, localLambda, geneLength,nominalP, fullyContained}

Exceptions
:   |  |  |
    | --- | --- |
    | IOException |  |

Here is the caller graph for this function:

|  |  |  |  |
| --- | --- | --- | --- |
| double [] broad.pda.seq.segmentation.ContinuousDataAlignmentModel.scoreGene | ( | Gene | *align*, |
|  |  | double | *localLambda* |
|  | ) |  | throws IOException |

|  |  |  |  |
| --- | --- | --- | --- |
| GeneScore broad.pda.seq.segmentation.ContinuousDataAlignmentModel.scoreGene | ( | Gene | *align*, |
|  |  | IntervalTree< Alignment > | *tree*, |
|  |  | double | *localLambda* |
|  | ) |  | throws IOException |

|  |  |  |  |
| --- | --- | --- | --- |
| GeneScore broad.pda.seq.segmentation.ContinuousDataAlignmentModel.scoreGene | ( | Gene | *align*, |
|  |  | IntervalTree< Alignment > | *tree*, |
|  |  | double | *localLambda*, |
|  |  | int | *numMarkers* |
|  | ) |  | throws IOException |

|  |  |  |  |
| --- | --- | --- | --- |
| double [] broad.pda.seq.segmentation.ContinuousDataAlignmentModel.scoreGene | ( | Gene | *align*, |
|  |  | IntervalTree< Alignment > | *tree* |
|  | ) |  | throws IOException |

|  |  |  |  |
| --- | --- | --- | --- |
| Map<Gene, double[]> broad.pda.seq.segmentation.ContinuousDataAlignmentModel.scoreGenes | ( | Collection< Gene > | *set*, |
|  |  | String | *chrToUse*, |
|  |  | IntervalTree< Alignment > | *tree* |
|  | ) |  | throws IOException |

Here is the call graph for this function:

Here is the caller graph for this function:

|  |  |  |  |  |  |
| --- | --- | --- | --- | --- | --- |
| Map<Gene, double[]> broad.pda.seq.segmentation.ContinuousDataAlignmentModel.scoreGenes | ( | Collection< Gene > | *genes* | ) | throws IOException |

Here is the call graph for this function:

|  |  |  |  |
| --- | --- | --- | --- |
| Map<Gene, double[]> broad.pda.seq.segmentation.ContinuousDataAlignmentModel.scoreGenes | ( | Collection< Gene > | *set*, |
|  |  | String | *chrToUse* |
|  | ) |  | throws IOException |

Here is the call graph for this function:

|  |  |  |  |
| --- | --- | --- | --- |
| Map<Gene, double[]> broad.pda.seq.segmentation.ContinuousDataAlignmentModel.scoreGenes | ( | Collection< Gene > | *set*, |
|  |  | String | *chrToUse*, |
|  |  | int | *size* |
|  | ) |  | throws IOException |

|  |  |  |  |  |  |
| --- | --- | --- | --- | --- | --- |
| void broad.pda.seq.segmentation.ContinuousDataAlignmentModel.scoreGenes | ( | BEDFileParser | *genes* | ) | throws IOException |

Here is the call graph for this function:

|  |  |  |  |
| --- | --- | --- | --- |
| Map<Path, double[]> broad.pda.seq.segmentation.ContinuousDataAlignmentModel.scorePaths | ( | Collection< Path > | *paths*, |
|  |  | double | *lambda* |
|  | ) |  | throws IOException |

Here is the call graph for this function:

Here is the caller graph for this function:

|  |  |  |  |
| --- | --- | --- | --- |
| static Map<Path, double[]> broad.pda.seq.segmentation.ContinuousDataAlignmentModel.scorePaths | ( | Collection< Path > | *paths*, |
|  |  | ChromosomeWithBubblesJGraphT | *graph*, |
|  |  | double | *alpha* |
|  | ) |  |  |

static

Here is the call graph for this function:

|  |  |  |  |
| --- | --- | --- | --- |
| static Map<Path, double[]> broad.pda.seq.segmentation.ContinuousDataAlignmentModel.scorePaths | ( | Collection< Path > | *paths*, |
|  |  | double | *lambda*, |
|  |  | double | *numMarkers*, |
|  |  | double | *numReads*, |
|  |  | double | *RPKMContant*, |
|  |  | Map< Annotation, Double > | *localRates*, |
|  |  | double | *alpha* |
|  | ) |  |  |

static

|  |  |  |  |  |  |
| --- | --- | --- | --- | --- | --- |
| Map<Path, double[]> broad.pda.seq.segmentation.ContinuousDataAlignmentModel.scorePathsForOnlyRPKM | ( | Collection< Path > | *paths* | ) |  |

|  |  |  |  |  |  |
| --- | --- | --- | --- | --- | --- |
| double [] broad.pda.seq.segmentation.ContinuousDataAlignmentModel.scoreSegment | ( | Annotation | *align* | ) | throws IOException |

|  |  |  |  |  |  |
| --- | --- | --- | --- | --- | --- |
| Map<Annotation, double[]> broad.pda.seq.segmentation.ContinuousDataAlignmentModel.scoreSegments | ( | Collection< Annotation > | *set* | ) | throws IOException |

|  |  |  |  |
| --- | --- | --- | --- |
| Map<Annotation, double[]> broad.pda.seq.segmentation.ContinuousDataAlignmentModel.scoreSegments | ( | Collection< Annotation > | *set*, |
|  |  | String | *chrToUse* |
|  | ) |  | throws IOException |

Here is the call graph for this function:

Here is the caller graph for this function:

|  |  |  |  |
| --- | --- | --- | --- |
| Map<Annotation, double[]> broad.pda.seq.segmentation.ContinuousDataAlignmentModel.scoreSegments | ( | Collection< Annotation > | *set*, |
|  |  | String | *chrToUse*, |
|  |  | boolean | *ignoreAlpha* |
|  | ) |  | throws IOException |

|  |  |  |  |  |  |
| --- | --- | --- | --- | --- | --- |
| void broad.pda.seq.segmentation.ContinuousDataAlignmentModel.setAlpha | ( | double | *alpha* | ) |  |

|  |  |  |  |  |  |
| --- | --- | --- | --- | --- | --- |
| void broad.pda.seq.segmentation.ContinuousDataAlignmentModel.setExtensionFactor | ( | int | *extensionFactor* | ) |  |

|  |  |  |  |  |  |
| --- | --- | --- | --- | --- | --- |
| void broad.pda.seq.segmentation.ContinuousDataAlignmentModel.setFindMaxContiguous | ( | boolean | *findMaxContiguous* | ) |  |

|  |  |  |  |  |  |
| --- | --- | --- | --- | --- | --- |
| void broad.pda.seq.segmentation.ContinuousDataAlignmentModel.setMaskFileData | ( | Map< String, Integer > | *maskedRegionMap* | ) |  |

|  |  |  |  |  |  |
| --- | --- | --- | --- | --- | --- |
| void broad.pda.seq.segmentation.ContinuousDataAlignmentModel.setMinContguousSegmentSize | ( | int | *minSize* | ) |  |

|  |  |  |  |  |  |
| --- | --- | --- | --- | --- | --- |
| void broad.pda.seq.segmentation.ContinuousDataAlignmentModel.setMinimumMappingQuality | ( | double | *minMapQual* | ) |  |

|  |  |  |  |  |  |
| --- | --- | --- | --- | --- | --- |
| void broad.pda.seq.segmentation.ContinuousDataAlignmentModel.setMinNumberOfSplices | ( | int | *minSpliceSupport* | ) |  |

|  |  |  |  |  |  |
| --- | --- | --- | --- | --- | --- |
| void broad.pda.seq.segmentation.ContinuousDataAlignmentModel.setTrimEnds | ( | boolean | *trimEnds* | ) |  |

|  |  |  |  |  |  |
| --- | --- | --- | --- | --- | --- |
| void broad.pda.seq.segmentation.ContinuousDataAlignmentModel.setTrimQuantile | ( | double | *quantile* | ) |  |

|  |  |  |  |  |  |
| --- | --- | --- | --- | --- | --- |
| void broad.pda.seq.segmentation.ContinuousDataAlignmentModel.setUpWeightSplices | ( | boolean | *setFlag* | ) |  |

|  |  |  |  |
| --- | --- | --- | --- |
| Gene broad.pda.seq.segmentation.ContinuousDataAlignmentModel.trimGeneEnds | ( | Gene | *gene*, |
|  |  | double | *quantile* |
|  | ) |  | throws IOException |

Here is the call graph for this function:

|  |  |  |  |
| --- | --- | --- | --- |
| Gene broad.pda.seq.segmentation.ContinuousDataAlignmentModel.trimMaxContiguous | ( | Gene | *region*, |
|  |  | double | *quantile* |
|  | ) |  | throws IOException |

Trim a region to a contiguous subregion with max number of positions having coverage above the quantile

Parameters
:   |  |  |
    | --- | --- |
    | region | The original region |
    | quantile | The coverage quantile of the original region |

Returns
:   The contiguous subregion with maximal number of positions having coverage above the quantile of the original region or null if can't trim

Exceptions
:   |  |  |
    | --- | --- |
    | IOException |  |

Here is the call graph for this function:

|  |  |  |  |
| --- | --- | --- | --- |
| void broad.pda.seq.segmentation.ContinuousDataAlignmentModel.writeConnections | ( | String | *save*, |
|  |  | ChromosomeWithBubblesJGraphT | *graph* |
|  | ) |  | throws IOException |

Here is the call graph for this function:

|  |  |  |  |
| --- | --- | --- | --- |
| static void broad.pda.seq.segmentation.ContinuousDataAlignmentModel.writeFullBED | ( | String | *save*, |
|  |  | Map< Gene, double[]> | *map*, |
|  |  | double | *alpha* |
|  | ) |  | throws IOException |

static

|  |  |  |  |
| --- | --- | --- | --- |
| static void broad.pda.seq.segmentation.ContinuousDataAlignmentModel.writeFullBED | ( | String | *save*, |
|  |  | Collection< Gene > | *segments* |
|  | ) |  | throws IOException |

static

## Member Data Documentation

double broad.pda.seq.segmentation.ContinuousDataAlignmentModel.DEFAULT\_INS\_SIZE\_PVAL = 0.05

static

int broad.pda.seq.segmentation.ContinuousDataAlignmentModel.DEFAULT\_INSERT\_SIZE\_FUDGE = 20

static

double broad.pda.seq.segmentation.ContinuousDataAlignmentModel.DEFAULT\_MAKE\_GENE\_OVERLAP = 0.3

static

int broad.pda.seq.segmentation.ContinuousDataAlignmentModel.DEFAULT\_MIN\_MAPPING\_QUALITY = 5

static

---

The documentation for this class was generated from the following file:

- java/broad/pda/seq/segmentation/ContinuousDataAlignmentModel.java


---

Generated on Fri Aug 15 2014 15:11:04 for ESAT by  

 1.8.7
